# Psycho-Cognitive Profile and NGF and BDNF Levels in Tears and Serum: A Pilot Study in Patients with Graves’ Disease

**DOI:** 10.3390/ijms24098074

**Published:** 2023-04-29

**Authors:** Alice Bruscolini, Angela Iannitelli, Marco Segatto, Pamela Rosso, Elena Fico, Marzia Buonfiglio, Alessandro Lambiase, Paola Tirassa

**Affiliations:** 1 Department of Sense Organs, University Sapienza of Rome, Viale del Policlinico 155, 00161 Rome, Italy; alice.bruscolini@uniroma1.it (A.B.); alessandro.lambiase@uniroma1.it (A.L.); 2 Department of Biotechnological and Applied Clinical Sciences, University of L’Aquila, Via Vetoio, Coppito 2, 67100 L’Aquila, Italy; iannitelliangela@gmail.com; 3 Department of Biosciences and Territory, University of Molise, Contrada Fonte Lappone, 86090 Pesche, Italy; marco.segatto@unimol.it; 4 Institute of Biochemistry & Cell Biology (IBBC), National Research Council (CNR), Unit of Translational & Biomolecular Medicine “Rita Levi-Montalcini”, Viale dell’Università 33, 00185 Rome, Italy; pamela.rosso@ibbc.cnr.it (P.R.); elena.fico@ibbc.cnr.it (E.F.); 5 Headache Center, Policlinico Umberto I, Sapienza University, Viale dell’Università 33, 00185 Rome, Italy; marziabuonfiglio@libero.it

**Keywords:** NGF, BDNF, Graves’ orbitopathy, CANTAB test, cognitive impairment, neuropsychiatric disorders

## Abstract

Nerve Growth Factor (NGF) and Brain derived Neurotrophic Factor (BDNF) mature/precursor imbalance in tears and serum is suggested as a risk factor and symptomatology aggravation in ophthalmology and neuropsychiatric disturbances. Cognitive and mood alterations are reported by patients with Graves’ Orbitopathy (GO), indicating neurotrophin alterations might be involved. To address this question, the expression levels of NGF and BDNF and their precursors in serum and tears of GO patients were analyzed and correlated with the ophthalmological and psycho-cognitive symptoms. Hamilton Rating Scale for Anxiety (HAM-A) and Depression (HAM-D), Temperament and Character Inventory (TCI), and Cambridge Neuropsychological Test Automated Battery (CANTAB) test were used as a score. NGF and BDNF levels were measured using ELISA and Western Blot and statistically analyzed for psychiatric/ocular variable trend association. GO patients show memorization time and level of distraction increase, together with high irritability and impulsiveness. HAM-A and CANTAB variables association, and some TCI dimensions are also found. NGF and BDNF expression correlates with ophthalmological symptoms only in tears, while mature/precursor NGF and BDNF correlate with the specific psycho-cognitive variables both in tears and serum. Our study is the first to show that changes in NGF and BDNF processing in tears and serum might profile ocular and cognitive alterations in patients.

## 1. Introduction

The Nerve Growth Factor (NGF) and Brain-Derived Neurotrophic Factor (BDNF) are expressed in human organs, including eyes, and a detectable amount of these neurotrophins (NTs) are measurable in human fluids, such as blood [1,2,3], saliva [4], and tears [5]. A consistent body of evidence demonstrates the clinical values of NGF and BDNF changes in serum by showing their correlation with the insurgence, severity, and response to treatments in neurological, immune, and psychiatric diseases [1,2,6].

In serum, such as in tissues, mature and precursor NGF and BDNF forms have been detected, and their imbalance is suggested as a risk factor for the aggravation of clinical symptoms, including cognitive and mood alterations, in diverse pathological conditions [1,7]. The expression of NGF and BDNF has been also largely described in ocular tissues and in tears of patients with ocular pathologies [8,9], but no studies have investigated the profile of NGF and BDNF and their precursors in serum and tears in correlation with ophthalmological and or no ophthalmological manifestations and symptoms of Graves’ disease (GD).

These questions have been addressed by this pilot study on a small group of patients affected by Graves’ Orbitopathy (GO), a typical ocular disorder affecting more than 50% of patients with the autoimmune GD [10].

GO is characterized by dry eye disease of varying severity associated with signs and symptoms of periorbital soft tissues inflammation such as palpebral and/or conjunctival chemosis and hyperemia, proptosis, lid retraction, and extraocular muscles oedema followed by progressive diplopia [11]. However, beside the ophthalmological symptoms, patients with GO might also present cognitive and mood disturbances [12]. The disfiguring proptosis and/or diplopia which are usually in women is often associated with anxiety and depression [13]. Emotional symptoms (impulsiveness and irritability), cognitive deficits (impairments in memory, concentration, attention, planning, and productivity), and affective disturbance (mania and depression) are also manifested by GD patients [13,14,15]. These disabling symptoms, which persist after normalization of hormonal dysfunction [16], may cause social and psycho-cognitive distress in GO patients and severely impact on their quality of life [12,13,17].

Because of these clinical features, GO patients appear as a suitable group of study for investigating the trend of NTs expression in tears and serum in relation with the ocular and psycho-cognitive profile.

In the present study, the evaluation of expression levels of NGF/proNGF and BDNF/proBDNF were performed by Western Blot (WB) analysis following protocols previously standardized in our laboratories to detect the mature and precursor NTs in ocular tissues and brains [18,19]. Ophthalmological evaluations, including proptosis and clinical activity score (CAS) and the psycho-cognitive status, evaluated by using Cambridge Neuropsychological Test Automated Battery (CANTAB) test, and the Hamilton scales plus the Temperament and Character Inventory (TCI), were applied to profile GO patients.

The results reported confirm the presence of proNGF and proBDNF in human serum and tears, and for the first time demonstrate the correlation between serum/tear NGF and BDNF, and their association with the patients’ ophthalmological and psycho-cognitive profile. The biological and clinical significance of these data are discussed, suggesting further multidisciplinary research involving cognitive behavioral and linked neurophysiological correlations.

## 2. Results

### 2.1. Psychiatric Evaluation

All the subjects included in the study did not manifest pathological signs of depression as evaluate by Hamilton scale (HAM-D). Only two GO patients present low levels of depression (HAMD: 8–17), but no changes were found between the groups (HC = 5.2 ± 1.4; GO = 7.41 ± 5.3). A significant increase in anxiety (HAM-A score) was manifested by GO patients (HC = 3.4 ± 2.6; GO = 12.4 ± 11.9; *p* = 0.026), but only one of them resulted high anxious (HAM-A > 34).

Based on CANTAB and TCI tests, selective alterations in cognitive performance and dimensions of personality traits were found in GO patients. The descriptive statistics and significances are reported as supplementary data in Tables S1 and S2, respectively.

The cognitive assessment shows significant changes in Paired Associates Learning (PAL) measures in GO patients, such as the total errors (PAL-TE *p* = 0.043), and the medial tentative (PAL-MT *p* = 0.004), the SWM Between Errors Total (SWM-BE *p* = 0.036), and in RVP-A-Prime (*p* = 0.0054) (Table S1).

The increased time in memorization, as well as the higher levels of distraction indicate a deficit in attention in GO patients. The significant correlation between the RVP-A and the anxiety rating found in GO patients (Pearson’s r = 0.530, *p* = 0.013) might justify this finding.

As far as the TCI is concerned, the main differences between the HC and the GO patients were found in the dimension of Reward Dependence (TCI-RD TOT, *p* < 0.001), and Cooperativeness (TCI-TOT, *p* < 0.001) (Table S2). In addition, GO patients showed a significant increase in impulsivity (TCI-NS2, *p* = 0.011) and irritability (TCI-P, *p* = 0.003). A correlation between HAM-A and TCI dimensions score in GO patients was also found (Table S3), indicating that mood status might influence the relationship with the social context.

### 2.2. Effects of GO on NGF and BDNF Serum

The data obtained by ELISA show that the NGF concentration is reduced in GO patients compared with HC (GO = 31.1 ± 17.79 pg/mL vs. HC =108.4 ± 87.22 pg/mL; F(1,24) = 17.08, *p* < 0.05), while no changes in BDNF are observable (GO = 31.61 ± 2.81 pg/mL vs. HC =34.2 ± 7.91 pg/mL; F(1,24) = 1.35).

**Figure 1:**
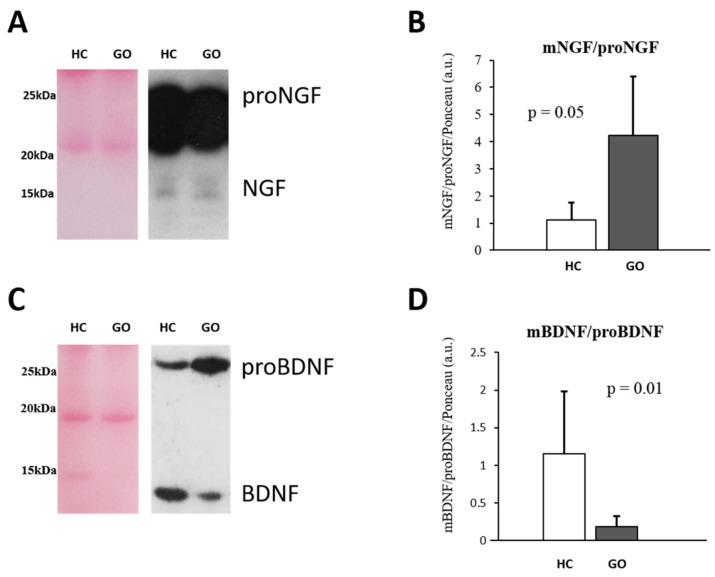
(**A**–**D**) WB analysis of NGF and BDNF in serum. Representative blots of NGF and BDNF bands detected in serum are shown in (**A**,**C**), respectively. Graphs B and D show the different expression ratio of mature and precursor NGF (**B**) and BDNF (**D**) in serum of HC and GO patients. Ponceau S staining was used to normalize sample-to-sample variability. Data are reported as ratio between mature and precursor neurotrophins, expressed as arbitrary units (a.u.) as described in the Section 4.

To further investigate potential NGF and BDNF level variations in serum, a WB analysis was performed, (Figure 1A–D). This shows that the relative expression of mature and precursor NTs is also affected by GO. A significant decrease in proNGF (GO = 0.369 ± 0.135 vs. HC = 1.0 ± 0.238; F(1,24) = 74.87, *p* < 0.001), but no difference in the NGF expression (GO = 1.59 ± 0.86 vs. HC =1.00 ± 0.33; F(1,24) = 3.26, *p* = 0.08) were found in patients with GO. The ratio NGF/proNGF is consequently increased in GO patients (GO = 4.21 ± 2.12 vs. HC = 1.12 ± 0.59; F(1,24) = 16.53, *p* = 0.05) (Figure 1A,B).

Within the GO group, the higher levels of NGF are detectable in group II (group I = 2.02 ± 0.9 vs. group II = 1.04 ± 0.45; *p* = 0.014) while no changes in proNGF expression levels were found between the two groups.

The comparison analysis of the data obtained by WB analysis shows a 4-fold decrease in BDNF levels (GO = 0.297 ± 0.21 vs. HC =1.10 ± 0.75; F(1,24) = 13.92, *p* < 0.05), and a little increase in proBDNF (GO = 1.679 ± 0.42 vs. HC = 1.33 ± 0.36; F(1,24) = 4.05, *p* < 0.05) in serum of GO patients with respect to HC. The BDNF/proBDNF ratio resulted significantly reduced in GO patients (*p* = 0.01) (Figure 1C,D). Taking the GO groups into consideration, in the group I the levels of BDNF (0.43 ± 0.17; *p* = 0.009) and proBDNF (2.06 ± 0.37; *p* = 0.02) were both increased compared with the group II (BDNF = 0.15 ± 0.12; proBDNF = 1.5 ± 0.24).

In our group of GO patients, no significant correlation between serum neurotrophins and T3 and T4 levels, as well as TRAb, were found.

### 2.3. Neurotrophin Expression in Tears of GO Patients

**Figure 2:**
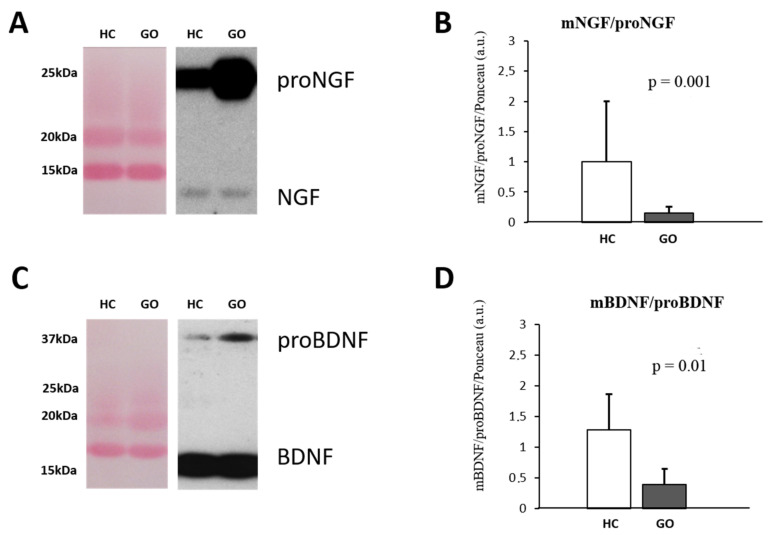
(**A**–**D**) WB analysis of NGF and BDNF in human tears. Representative blots of NGF and BDNF bands detected in tears are shown in (**A**,**C**), respectively. Graphs B and D show the different expression ratio of mature and precursor NGF (**B**) and BDNF (**D**) in tears of HC and GO patients. Ponceau S staining was used to normalize sample-to-sample variability. Data are reported as ratio between mature and precursor neurotrophins, expressed as arbitrary units (a.u.) as described in the Section 4.

Parallel to what it is observable in serum, in tears also the larger detectable NGF form is the proNGF (Figure 2A–D), and the WB analysis reveals an increase in proNGF (GO = 9.46 ± 6.49 vs. HC = 1.10 ± 0.494; F(1,18) = 12.99 *p* < 0.05) and no variance of NGF (GO = 0.898 ± 0.267 vs. HC = 1.15 ± 0.580; F(1,18) = 1.75 *p* = 0.54) expression levels in the tears of GO patients. A significant decrease in the NGF/proNGF ratio (GO = 0.149 ± 0.109 vs. HC = 0.931 ± 0.432; F(1,18) = 37.53, *p* = 0.001) was thus found in GO patients’ tears (Figure 2A,B). Similar to NGF, the comparative analysis shows that in tears of GO patients, the levels of proBDNF are about 3 times higher than those detected in HC (GO = 3.75 ± 2.0 vs. HC = 1.06 ± 0.678; F(1,18) = 13.92, *p* < 0.05), while no changes in mature form were found (GO = 0.93 ± 0.35 vs. HC = 1.0 ± 0.108; F(1,18) = 0.266, *p* = 0.82). The BDNF/proBDNF ratio in GO was significantly decreased with respect to HC (GO = 0.323 ± 0.17 vs. HC = 1.27 ± 0.62; F(1,18) = 25.46, *p* = 0.01) (Figure 2C,D).

Within the GO groups, decreased levels of tears BDNF (0.62 ± 0.02; *p* < 0.001) and proBDNF (2.9 ± 1.7; *p* = 0.073) were found in group II compared with the group I (BDNF = 1.2 ± 0.17; proBDNF = 4.7 ± 1.7). An increase in the NGF and proNGF levels, although it does not reach the significant levels, were found in the group II compared group I.

**Figure 3:**
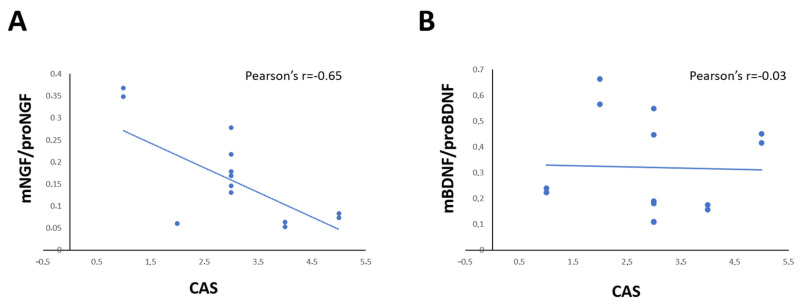
(**A**,**D**) Graphs show the correlation between the ratio of mNGF/proNGF (**A**) and mBDNF/proBDNF (**B**) expression in tears and CAS score in GO patients. Data are reported as ratio between mature and precursor neurotrophins, expressed as arbitrary units (a.u.) as described in the Section 4.

The correlation analysis showed that the levels of NGF and proNGF in tears of each GO patient is correlated in positive manner with proptosis (Pearson’s r = 0.748; *p* = 0.002; Spearman’s rho = 0.927 *p* < 0.001), and CAS (Pearson’s r = 0.874; *p* < 0.001; Spearman’s rho = 0.750 *p* < 0.002), but the NGF/proNGF is significantly negatively correlated with CAS only (Pearson’s r = 0.643; *p* = 0.013) (Figure 3A). A negative correlation between BDNF and both proptosis (Pearson’s r= −0.616 *p* = 0.019; Spearman’s rho = −0.500 *p* = 0.069) and CAS (Pearson’s r = 0.602; *p* = 0.023; Spearman’s rho = −0.371 *p* = 0.192), but no correlation between BDNF/proBDNF and CAS (Pearson’s r = 0.031; *p* = 0.091; Spearman’s rho = −0.230 *p* = 0.429) was found (Figure 3B).

Patient’s CAS score was also positively correlated with serum NGF/proNGF (Pearson’s r = 0.602; *p* = 0.023; Spearman’s rho = −0.371 *p* = 0.192) levels, but not with serum BDNF/proBDNF levels (Pearson’s r = −0.189; *p* = 0.557; Spearman’s rho = −0.116 *p* = 0.720).

### 2.4. Psycho-Cognitive and NT Profile in GO Patients

In line with the comparative analysis reported in paragraph 2.1, which demonstrated a selective deficit in GO in Paired Associates Learning (PAL) and Rapid Visual Information Processing (RVP), the regression analysis shows a significant correlation between the scores of these parameters and the NGF and BDNF expression in tears and serum (* *p* < 0.1, ** *p* < 0.01, *** *p* < 0.001).

As it is shown in Table S4, both serum and tears of m/proNGF ratio are negatively correlated with the PAL scores (PAL – TE, Pearsons’s r: serum −0.536 * and tears −0.692 **, and Spearman’s rho: serum −0.609 ** and tears −0.594; and PAL – MT, Pearsons’s r: serum −0.617 ** and tears −0.642 **, and Spearman’s rho: serum 0.750 *** and tears −0.642 *), so that the higher scores are present in association with low levels of NGF (or high levels of proNGF) in the two fluids.

On the contrary, both PAL scores are positively correlated with the m/proBDNF ratio in tears and serum (PAL – TE, Pearsons’s r: serum 0.665 ** and tears 0.712 **, and Spearman’s rho: tears 0.559 *; and PAL - TE6, Pearsons’s r: serum 0.849 *** and tears 0.683 **, and Spearman’s rho: serum 0.714 ** and tears 0.751 *), indicating the accuracy and latency increase parallel with the increase in BDNF (or decline of proBDNF). A negative correlation was found between the RVP - ML scores and the m/proBDNF levels in both serum and tear (Pearsons’s r: serum −0.631 * and tears −0.580 *, and Spearman’s rho: serum 0.714 ** and tears −0.750 **) (Table S4).

A far as the TCI is concerned, the correlation analysis between the TCI score and NT levels in GO patients shows differences between the serum and tears for both NTs, as reported in Table S5. A negative correspondence between the serum m/proBDNF and NS1, and RD2, and C traits was found (NS1, Pearsons’s r: serum −0.879 ***, and Spearman’s rho: serum −0.812 **; RD2, Pearsons’s r: serum −0. 947 ***, and Spearman’s rho: serum −0.971 ***), while the serum m/proNGF is negatively correlated with NS2, and T3 and T TOT traits (NS2, Pearsons’s r: serum −0.706 ***, and Spearman’s rho: serum −0.653 **; T3, Pearsons’s r: serum −0.530 *, and Spearman’s rho: serum −0.580 *; and T TOT, Pearsons’s r: serum −0.460 *, and Spearman’s rho: serum −0.433), but positively related to HA (Pearsons’s r: serum −0.758 ***, and Spearman’s rho: serum −0.726 ***), and SD2 (Pearsons’s r: serum −0.475 *, and Spearman’s rho: serum −0.650 **).

Only a few traits were found to correlate with both NT ratio in serum and tears. Specifically, NS1 and RD2 traits correlated with BDNF ratio (NS1 Pearsons’s r: serum −0.879 *** and tears −0.779 **, and Spearman’s rho: serum −0.812 ** and tears −0.703 **; and RD2, Pearsons’s r: serum −0. 947 *** and tears −0.514 *, and Spearman’s rho: serum −0.971 *** and tears −0.505), while NS2 (Pearsons’s r: serum −0.706 *** and tears −0.688 **, and Spearman’s rho: serum −0.653 ** and tears −0.545 *) and T traits (T3, Pearsons’s r: serum −0.530 * and tears −0.790 ***, and Spearman’s rho: serum −0.508 * and tears −0.666 **and T TOT: Pearsons’s r: serum −0.460 * and tears −0.877 ***, and Spearman’s rho: serum −0.433 and tears −0.749 **) correlated with NGF ratio, as indicated in bold in Table S5.

## 3. Discussion

The NGF and BDNF serum and tear profile was investigated in HC and GO patients by measuring the levels of NTs using commercial kits which recognize human NGF and BDNF, and WB analysis to detect the mature and precursor forms. The obtained results extend the previous observations that tear and serum NGF concentrations are altered in Graves’ patients [20,21], showing the contemporaneous changes in NGF and BDNF levels, and the expression trend of the mature and precursor NTs. Moreover, our findings suggest the potential application of serum and tear NTs as biomarkers for the cognitive/mood aspects of GO diseases.

The parallel measure of serum NGF and BDNF expression by ELISA and WB allow us to identify a correspondence between the individual NT concentration measured by the commercial immunoassay kits and the mature/proNT ratio. We found that ELISA BDNF and NGF concentrations are decreased and unchanged in GO patients, respectively, while the WB analysis shows a reduction in proNGF, but no NGF, and an increase in proBDNF in the presence of a low expression of BDNF. As observed by Malerba et al. [22] and supported by our previous studies [18], the ELISA kits, even if specific for the NTs, measure the total (mature plus precursors) amount of NGF and BDNF, and their results might be affected by high levels of precursors. Thus, the reduction in NGF found by ELISA in the serum GO, where the level of precursor is several times higher than the mature form, might actually be due to the proNGF, while the contemporaneous increase in proBDNF and decrease in BDNF might justify the lack of difference in the BDNF concentrations detected by ELISA.

Moreover, our WB analysis shows that NGF and BDNF, and their precursors, are expressed in the tears. Like in serum, and to what has been found previously in other tissues [22], the proNGF is the most expressed form in human tears, while high BDNF and low proBDNF levels were detectable in the tears of HC. The finding that the relative expression of mature/pro NGF and BDNF in the two fluids follows an opposite trend, suggests that different mechanisms might occur to regulate the ocular and circulating NGF and BDNF processing and release.

In this context, we found an increase in proNGF and proBDNF, but not changes in mature forms in GO tears compared with HC. Since the levels of NGF and BDNF in tears depend mainly on their synthesis by lacrimal grands [23], it is likely that the NT imbalance observed might be directly associated with a GO-induced alteration in local processing and/or release. The evidence that structural changes in lacrimal glands associated with ocular surface alterations are reported in Graves’ diseases [24], as in other pathologies with thyroid dysfunction [25], might support our suggestion. Our hypothesis might also be strengthened by the findings that the expression of NGF, but not BDNF, correlate with the CAS and proptosis score in the opposite manner. In line with this, the findings that the patients’ NGF/proNGF level in both tears and serum, but not BDNF/proBDNF, correlates with their CAS score, even if only with moderate linear regression in tears, might support the clinical value of mature and precursor ratio as pathological marker in ophthalmology, suggesting the need to further investigate the changes in neurotrophin forms in a larger patient population.

The clinical values of NT changes in tears in GO ophthalmological manifestations, which include conjunctival inflammation and eye dryness, is also in accordance with the evidences that the local production of NTs in eyes and their release in tears are crucial for the maintenance of healthy ocular surfaces, and exert a neuroprotective and anti-inflammatory role in ocular pathologies and dry eye [26].

However, it cannot even be ruled out that NT imbalance in tears might have been already present in the preclinical phase and/or have a role in the manifestation of other symptoms reported in GO, such as memory impairment and mood disturbances, and psychosocial functions [13].

Changes in NGF and BDNF levels in serum have been detected in patients with neurodegenerative and psychiatric diseases, anxiety, mood disorders, and stress [6,19,27] but the importance of distinguishing mature and precursor NTs has been pointed out only recently. Very little is known about the serum NGF and proNGF in humans, while the serum BDNF and proBDNF ratio has been investigated—, also the relationship to the psycho-cognitive parameters, in patients with major depression, obstructive sleep apnea with psoriasis, and autism [28,29,30,31]. No studies have addressed the questions about the expression of NT forms in tears, and to the best of our knowledge, our study is the first to investigate the psycho-cognitive parameters in patients and the contemporaneous changes in m/pro NT ratio in serum and tears.

Chiefly, we found that impairment of episodic memory in GO, as indicated by increased PAL scores, is associated with the tear and serum NGF and BDNF m/pro expression in a negative (low NGF levels) and positive manner (high BDNF levels), respectively, while only m/pro BDNF correlated with RVP.

Because of the novelty of our data, the significance of these correlations can be only speculated. For example, it is interesting that all PAL tasks correlated with tear NGF and BDNF, while only some tasks correlated with serum. One possible explanation might arise from the evidence that visual memory is coupled with increased eye movements and blinking [32,33], and that the eye blinking rate is an index of emotion and attention [34], and dopamine brain pathway [35]. Spontaneous blinking, as well as eye movement and tear secretion, are affected in GO patients [36], it is thus possible that the NTs in tears, in particular NGF, might represent the molecular corresponding of the eye blink-based prediction of cognitive/dopamine alterations in GO patients. To explore this hypothesis, a new study involving GO patients is addressed to evaluate the blink rate, as well as the dopamine/serotonin levels in tears, and investigate the correlation with the NT profile.

The specific correlation between m/pro BDNF in both serum and tears with RVP might corroborate the involvement of the eye–brain pathway. Indeed, BDNF is known to be directly regulated by the visual stimulation [37,38,39,40,41], and its processing and release, also in serum, is activity dependent, and susceptible of changes in stress and in pathological conditions [42]. Moreover, low BDNF levels are reported in patients with cognitive deficits often associated with depression and anxiety [43], and subjects with BDNF Val66Met polymorphism (rs6265), which affects proBDNF processing in mature forms, shows poorer visual memory [44], and a high risk of developing mood disorders [45].

In this study, we found that in GO patients, the RVP score correlated with the HAM-A value indicating that the attention impairment is associated with anxiety. The correlation between anxiety and impulsivity, as well as irritability, further support the idea that mood status and increased stress sensitivity and/or reactivity might characterize GO patients. It is therefore possible that the correlation between m/pro BDNF and the RVP we found in the serum and tears of GO patients might be also representative of the emotional dimension of disease, and therefore impact on their ability to maintain attention.

## 4. Materials and Methods

### 4.1. Study Design and Patient’s Recruitment

This observational study was addressed to analyze the daily NGF and BDNF profiles in the tears and serum of healthy women and in patients with GO. This study was performed at the Department of Sense Organs of Sapienza University of Rome.

Ten healthy subjects (HC) and Twenty patients with GO were enrolled by an ophthalmologist with the following inclusion criteria: age older than 18 years, laboratory diagnosis of GD based on the finding of undetectable serum TSH and high blood thyroid hormone associated with the presence of circulating TSH receptors antibodies (TRAb), and signs and symptoms of GO according with EUGOGO guidelines [46]. Clinical history was collected and all patients underwent a complete eye examination including: (i) visual acuity evaluation by Early Treatment Diabetic Retinopathy (ETDR) charts; (ii) exophthalmometry with Hertel instrument; (iii) assessment of severity of diplopia using Gorman score (0: no diplopia; 1: intermittent diplopia, 2: inconstant diplopia and 3: constant diplopia) [47]; (iv) and assessment of GO activity with CAS and severity assessment according to EUGOGO classification (Mild GO = 1, Moderate-to-severe GO = 2, Sight-threatening GO = 3) [46]. A detailed description of the ocular evaluation in patients is reported as supplementary data, Table S6.

Ten healthy subjects, age matched, attending the outpatients’ service of the Eye Clinic of University of Rome “Sapienza”, were recruited as a control group by an ophthalmologist and were reported to not have signs and symptoms of GD and GO, to not take regular medications or drugs, to not have history of alcohol/drug addiction, to not be heavy alcohol drinkers (according to the indications of the National Institute on Alcohol Abuse and Alcoholism, NIAAA), and to not have drank alcoholic beverages in the past 42 h. Women were included in the first week of their post-menstrual period. All the subjects were not family related, did not report any personal or familiar neurological or psychiatric diseases, or allergic, infective, nor inflammatory disorders. All GO patients and healthy controls were referred to psychiatrists for valuations, as described below.

### 4.2. Psychiatric Evaluation

All subjects included in the study underwent a psychiatric interview (AI), and were tested for scoring their emotional and cognitive profile.

Patients’ mood conditions were assessed using the Hamilton Rating Scale for Anxiety (HAM-A) and Depression (HAM-D) [48]. The HAM-A scale consists of 14 items, each defined by a series of symptoms, and measures both psychic anxiety (mental agitation and psychological distress) and somatic anxiety (physical complaints related to anxiety). Each item is scored on a scale of 0 (not present) to 4 (severe), with a total score range of 0–56, where <17 indicates mild severity, 18–24 mild to moderate severity, and 25–30 moderate to severe.

The HAM-D contains 17 items (HDRS17) pertaining to symptoms of depression experienced over the past week. A score of 0–7 is generally accepted to be within the normal range (or in clinical remission), while a score of 20 or higher indicates at least moderate severity.

To assess the cognitive function, tasks from the Cambridge Neuropsychological Test automated Battery (CANTAB eclipse version 3.0; http://www.camcog.com) were administered to all participants by trained individuals (AQ). The CANTAB is a computer-administered, nonverbal (visually presented) set of tasks, developed to examine specific components of cognition, particularly those associated with frontal and medial temporal regions of the brain [49,50]. The testing session lasted approximately 90–120 min, with the duration of each test ranging 7–20 min. The CANTAB tests employed were: Paired Associates Learning (PAL) (episodic memory and learning) [51]; Spatial Working Memory (SWM) (working memory capacity and strategy use) [52]; and Rapid Visual Information Processing (RVP) (sustained attention) [53]. Descriptions of CANTAB tasks are presented in Table S7 [49,51,52,54].

Assessment of personality was performed by the Temperament and Character Inventory (TCI) developed by C. Robert Cloninger [55], based on four temperaments (Novelty Seeking (NS), Harm Avoidance (HA), Reward Dependence (RD), and Persistence (PS)) and three characters (Self-directedness (SD), Cooperativeness (CO), and Self-transcendence (ST)) each of which corresponds to a specific pattern of behavior in response to various environmental stimuli. Description of TCI tasks are presented as supplementary data (Table S8).

### 4.3. Sample Collection and Evaluation

Tears and blood were collected at 10:30 a.m. on the same day of the psychiatric interview. Tear samples were collected in both eyes from all patients by imbibition of a sterile Sharp-tip Microsponges (Alcon Lab. Inc., Fort Worth, TX, USA) placed at the inferior conjunctival fornix and removed after 30 s. Tears from the eyes of each patient were pooled, and after centrifugation at 13,000 rpm for 3 min, were stored at −20 °C until processing for WB analysis.

Approximately 10 mL of blood was drawn from the subject’s antecubital vein and left at room temperature until clot forming and clear serum was obtained by centrifugation. All samples were aliquoted and stored at –70 °C until use.

### 4.4. ELISA Detection of Neurotrophins

NGF and BDNF concentrations in serum and saliva were measured by using human NGF and BDNF immunoassay (DY256 for NGF and DY248 for BDNF, R&D system, Minneapolis, MN, USA). All the assays were performed in triplicate, following the manufacturer’s instruction and using the recommended buffers, diluents, and substrates. The optical density of the color reaction was read using a microtiter plate reader (Dynatech MR5000; PBI International, Dynatech International, Edgewood, NY, USA) set for 450 nm. The intra- and inter-assay coefficients of variation were below 7%. The NT concentration (expressed as pg/mL) in each sample was calculated according to a standard curve.

### 4.5. Western Blot Analysis

The expression levels of mature precursor forms of NGF and BDNF were analyzed by WB in tears and serum samples. Briefly, equal protein amounts were loaded on a 10% SDS-PAGE gel and electrophoretically transferred to nitrocellulose membranes later blocked in low-fat milk (10% in Tris buffer and 0.1% Tween-20) and washed three times for 10 min each at RT in TBS-T. Samples were exposed to the primary anti-NGF (1:1000, sc-365944, Santa Cruz Biotechnology, Dallas, TX, USA) and anti-proBDNF (1:1000, sc-65514, Santa Cruz Biotechnology, Dallas, TX, USA) antibodies for 1 h at room temperature (RT) on a shaker.

The blots were washed and incubated for 1 h with anti-rabbit or anti-mouse horseradish peroxidase conjugated antibody (Biorad, California, USA, #1706515 and #1706516, respectively) at RT on a shaker. Immunoblot analyses were performed using a chemiluminescence detection kit (ECL) as the chromophore (ECL system RPN2232 Amersham Bioscience, UK) and exposed to Hyperfilm (Amersham Hyperfilm ECL 28906837, Amersham, UK). The integrate density (ID) of the bands was recorded and quantified by the public domain image Fiji (2.9.0, ImageJ, NIH, Baltimore) Image processing and analysis program. Data are expressed as arbitrary units (a.u.).

### 4.6. Statistical Analysis

Statistical analysis was performed by using the free open-source program for statistical analysis JASP (0.17.1, Jeffreys’s Amazing Statistics Program) by the University of Amsterdam [56]. Based on data obtained in previous studies analyzing the BDNF and NGF levels in human serum and tears [27], a sample size of 20 patients was considered sufficient to obtain 80% statistical power to detect significant difference at α = 0.05. The variable distribution was assessed by the Shapiro–Wilk test. Statistical analysis of the psychiatric score and NT expression levels was conducted using the Student T test and ANOVA followed by Tukey’s post-hoc testing. All data are presented as the mean ± S.D. The significance level was set at *p* ≤ 0.05. The correlations analysis between the levels of NTs in serum and tears, as well as between the NTs and the psychiatric and ocular variables, were determined by both Pearson’s and Spearman correlation, as suggested by the Rovetta’s study [57]. Again, the significance level was set at *p* ≤ 0.05.

## 5. Conclusions

In conclusion, the present study is one of the first to explore the relationship between the NGF and BDNF expression in serum and tears of humans, and to show that altered ratio of mature and precursor NTs in these fluids correspond to the ophthalmological symptoms (proptosis and CAS), and the psycho-cognitive profile of GO patients. These observations enlarge the information about NT involvement in ocular diseases, as well as neuropsychiatric disturbances, and open a new insight into the validation of tears and serum NTs as clinical and therapeutical biomarkers.

## Data Availability

The data presented in this study are available on request from the corresponding author.

## Supplementary Materials

The supporting information can be downloaded at: https://www.mdpi.com/article/10.3390/ijms24098074/s1.
