# Supplementary material for: Psycho-Cognitive Profile and NGF and BDNF Levels in Tears and Serum: A Pilot Study in Patients with Graves’ Disease
Source: Int J Mol Sci. 2023 Apr 29;24(9):8074. doi: 10.3390/ijms24098074 (PMC10178719; doi:10.3390/ijms24098074)
Supplement: Supplementary file 1 [file ijms-24-08074-s001.zip › ijms-2301193-supplementary.pdf]

Supplementary data

Table S1. CANTAB descriptive statistics.

	Group	Mean	SD	T-test		
				t	df	p
**PAL-TE** (Total Error adjusted)	HC (n=10)	10.2	3	-2.115	28	**0.043**
	GO (n=20)	19.4	13.4			
**PAL-TE6** (TE six shapes adjusted)	HC	3.2	1.8	-1.806	28	0.082
	GO	7.5	7.3			
**PAL – FT** (First trial memory score)	HC	17.0	1.6	0.084	28	0.0933
	GO	16.9	3.5			
**PAL – MT** (mean of Tentative)	HC	1.3	0.1	-3.108	28	**0.004**
	GO	1.9	0.6			
**SWM - BE** (Between errors Total)	HC	14.8	5.7	-2.206	28	**0.036**
	GO	26.0	15.4			
**SWM - S** (Strategy)	HC	33.6	4.8	0.411	28	0.684
	GO	32.8	5.1			
**RVP -A**	HC	0.9	0.03	3.029	28	**0.005**
	GO	0.8	0.03			
**RVP - ML** (Mean latency)	HC	500.0	94.5	1.230	28	0.229
	GO	459.4	82.1			

Table S2. TCI descriptive statistics.

Temperaments							*Characters*						
	Group	Mean	SD	T-test t	df	p		Group	Mean	SD	T-test t	df	p
**TCI-NS1**	HC	5.800	2.616	-1.05	28	0.323	**TCI-SD1**	HC	7.400	0.516	1.33	28	0.192
	GO	6.800	2.546					GO	6.800	1.361			
**TCI-NS2**	HC	2.810	1.529	-2.74	28	**0.011**	**TCI-SD2**	HC	5.200	1.229	0.92	28	0.362
	GO	5.500	2.911					GO	4.600	1.847			
**TCI-NS3**	HC	5.000	1.333	1.39	28	0.174	**TCI-SD3**	HC	3.600	1.430	-1.74	28	0.092
	GO	4.000	2.052					GO	4.400	1.046			
**TCI-NS4**	HC	4.000	0.667	-0.95	28	0.346	**TCI-SD4**	HC	6.200	1.033	0.58	28	0.561
	GO	4.810	2.609					GO	5.600	3.119			
**TCI-NS TOT**	HC	17.60	3.373	-1.40	28	0.170	**TCI-SD5**	HC	10.40	1.838	1.39	28	0.173
	GO	21.10	7.433					GO	9.200	2.375			
**TCI-HA1**	HC	3.800	2.044	-0.62	28	0.534	**TCI-SD-TOT**	HC	32.80	2.150	1.01	28	0.319
	GO	4.300	2.055					GO	30.50	6.947			
**TCI-HA2**	HC	4.400	1.430	0.41	28	0.679	**TCI-C1**	HC	7.000	1.155	1.43	28	0.163
	GO	4.100	2.024					GO	5.900	2.269			
**TCI-HA3**	HC	3.800	2.044	1.55	28	0.131	**TCI-C2**	HC	6.800	0.422	5.14	28	**< .001**
	GO	2.605	1.951					GO	4.100	1.619			
**TCI-HA4**	HC	2.400	0.843	0.21	28	0.831	**TCI-C3**	HC	7.400	0.843	3.24	28	**0.003**
	GO	2.225	2.482					GO	6.200	1.005			
**TCI-HA TOT**	HC	14.40	5.797	0.53	28	0.597	**TCI-C4**	HC	9.400	1.265	1.69	28	0.101
	GO	13.20	5.800					GO	8.200	2.042			
**TCI-RD1**	HC	7.800	1.229	1.90	28	0.068	**TCI-C5**	HC	8.200	0.422	3.24	28	**0.003**
	GO	6.600	1.789					GO	7.000	1.124			
**TCI-RD2**	HC	7.000	0.943	4.60	28	**< .001**	**TCI-C TOT**	HC	38.80	2.044	3.69	28	**< .001**
	GO	4.300	1.720					GO	31.300	6.208			
**TCI-RD3**	HC	4.400	1.075	3.56	28	**0.001**	**TCI-ST1**	HC	4.400	2.459	-1.29	28	0.207
	GO	2.800	1.196					GO	5.900	3.227			
**TCI-RD TOT**	HC	19.20	2.348	4.06	28	**< .001**	**TCI-ST2**	HC	3.200	1.229	-2.20	28	**0.036**
	GO	13.70	3.922					GO	4.900	2.269			
**TCI-P**	HC	2.600	1.713	-3.24	28	**0.003**	**TCI-ST3**	HC	2.400	1.430	-0.39	28	0.694
	GO	5.000	2.000					GO	2.700	2.155			
							**TCI-ST-TOT**	HC	10.00	2.749	-1.59	28	0.122
								GO	13.50	6.605			

Table S3. Correlation between HAM-A and TCI dimensions in GO patients

TCINS1	Pearson's r	0.386	TCIRD1	Pearson's r	0.617**	TCIC1	Pearson's r	−0.221
	p-value	0.093		p-value	0.004		p-value	0.350
TCINS2	Pearson's r	0.524*	TCIRD2	Pearson's r	0.666**	TCIC2	Pearson's r	0.085
	p-value	0.018		p-value	0.001		p-value	0.723
TCINS3	Pearson's r	0.463*	TCIRD3	Pearson's r	0.355	TCIC3	Pearson's r	0.241
	p-value	0.040		p-value	0.125		p-value	0.305
TCINS4	Pearson's r	0.546*	TCIRDTOT	Pearson's r	0.682***	TCIC4	Pearson's r	0.263
	p-value	0.013		p-value	< .001		p-value	0.262
TCINSTOT	Pearson's r	0.658**	TCIP	Pearson's r	−0.240	TCIC5	Pearson's r	0.433
	p-value	0.002		p-value	0.308		p-value	0.057
TCIHA1	Pearson's r	−0.072	TCISD1	Pearson's r	0.171	TCICTOT	Pearson's r	0.159
	p-value	0.764		p-value	0.471		p-value	0.504
TCIHA2	Pearson's r	0.048	TCISD2	Pearson's r	0.320	TCIT1	Pearson's r	0.192
	p-value	0.842		p-value	0.169		p-value	0.418
TCIHA3	Pearson's r	−0.618**	TCISD3	Pearson's r	0.434	TCIT2	Pearson's r	−0.047
	p-value	0.004		p-value	0.056		p-value	0.844
TCIHA4	Pearson's r	−0.138	TCISD4	Pearson's r	−0.546*	TCIT3	Pearson's r	0.040
	p-value	0.563		p-value	0.013		p-value	0.868
TCIHATOT	Pearson's r	−0.274	TCISD5	Pearson's r	−0.463*	TCITTOT	Pearson's r	0.090
	p-value	0.243		p-value	0.040		p-value	0.704
			TCISDTOT	Pearson's r	−0.215			
				p-value	0.363			

* p < .05, ** p < .01, *** p < .001

Table S4. Correlation between NTs expression and CANTAB scores in GO patients.

		m/proBDNF		m/proNGF	
		Serum	Tears	Serum	Tears
**PAL - TE**	*Pearsons's r*	**0.665****	**0.712****	**-0.536***	**-0.692****
	p-value	**0.009**	**0.002**	**0.015**	**0.003**
	Spearman's rho	**0.371**	0.559*	**-0.609****	**-0594****
	p-value	**0.235**	0.038	**0.002**	**0.025**
**PAL - TE6**	*Pearsons's r*	**0.849*****	**0.683****	-0.346	-0.681**
	p-value	**< .001**	**0.004**	0.136	0.004
	Spearman's rho	**0.714****	**0.751***	-0.400	-0.892***
	p-value	**0.009**	**0.033**	0.100	< .001
**PAL - FT**	*Pearsons's r*	-0.198	-0.654**	**0.725*****	**0.865*****
	p-value	0.498	0.006	**< .001**	**< .001**
	Spearman's rho	-0.429	-0.679**	**-0.713****	**-0.655****
	p-value	0.165	0.008	**0.004**	**0.003**
**PAL - MT**	*Pearsons's r*	0.457	0.632**	**-0.617****	**-0.642****
	p-value	0.100	0.009	**0.004**	**0.007**
	Spearman's rho	0.371	0.536*	**0.750*****	**-0.642***
	p-value	0.235	0.048	**< .001**	**0.013**
**SWM - BE**	*Pearsons's r*	0.337	0.358	0.103	0.115
	p-value	0.239	0.173	0.666	0.672
	Spearman's rho	0.314	0.536*	-0.200	0.071
	p-value	0.320	0.048	0.426	0.808
**SWM - S**	*Pearsons's r*	0.112	0.437	0.083	-0.335
	p-value	0.703	0.090	0.728	0.204
	Spearman's rho	0.145	0.306	-0.181	-0.504
	p-value	0.653	0.287	0.463	0.066
**RVP - A**	*Pearsons's r*	-0.881***	-0.162	-0.173	0.217
	p-value	< .001	0.549	0.465	0.420
	Spearman's rho	-0.928***	-0.360	-0.134	0.252
	p-value	< .001	0.206	0.595	0.385
**RVP - ML**	*Pearsons's r*	**-0.631***	**-0.580***	-0.223	-0.259
	p-value	**0.015**	**0.018**	0.344	0.332
	Spearman's rho	**-0.714****	**-0.750****	-0.133	-0.071
	p-value	**0.009**	**0.002**	0.598	0.898

* p < .05, ** p < .01, *** p < .001.

Table S5. Correlation between NTs expression and TCI scores in GO patients.

		m/proBDNF		m/proNGF	
		Serum	Tears	Serum	Tears
TCI – NS1	*Pearsons's r*	**-0.879*****	**-0.779****	-0.132	0.225
	p-value	**< 0.001**	**0.001**	0.578	0.401
	Spearman's rho	**-0.812****	**-0.703****	0.034	0.180
	p-value	**0.001**	**0.005**	0.894	0.538
TCI – NS2	*Pearsons's r*	-0.206	0.461	**-0.706*****	**-0.688****
	p-value	0.521	0.097	**< 0.001**	**0.003**
	Spearman's rho	-0.265	0.273	**-0.653****	**-0.545***
	p-value	0.406	0.345	**< 0.003**	**0.044**
TCI – NS3	*Pearsons's r*	-0.648*	-0.302	-0.171	-0.303
	p-value	0.023	0.295	0.470	0.255
	Spearman's rho	-0.551	-0.327	-0.017	-0.109
	p-value	0.063	0.253	0.947	0.711
TCI – NS4	*Pearsons's r*	-0.085	-0.159	-0.331	-0.170
	p-value	0.792	0.588	0.180	0.562
	Spearman's rho	-0.116	-0.218	-0.136	-0.309
	p-value	0.720	0.454	0.590	0.283
TCI – NS TOT	*Pearsons's r*	-0.802***	-0.030	-0.461	-0.384
	p-value	< 0.001	0.912	0.054	0.141
	Spearman's rho	-0.771**	-0.199	-0.326	-0.384
	p-value	< 0.003	0.495	0.186	0.141
TCI – HA1	*Pearsons's r*	-0.263	0.214	0.012	-0.462
	p-value	0.363	0.432	0.961	0.071
	Spearman's rho	0.029	0.200	-0.068	-0.381
	p-value	0.929	0.493	0.788	0.178
TCI – HA2	*Pearsons's r*	-0.112	0.043	0.758***	0.154
	p-value	0.702	0.875	< 0.001	0.568
	Spearman's rho	-0.058	0.180	0.726***	-0.090
	p-value	0.858	0.538	< 0.001	0.760
TCI – HA3	*Pearsons's r*	0.801***	0.370	0.342	-0.464
	p-value	<0.001	0.158	0.140	0.070
	Spearman's rho	0.754**	0.306	0.417	-0.684**
	p-value	0.005	0.287	0.085	0.007
TCI – HA4	*Pearsons's r*	-0.315	-0.204	0.099	-0.433
	p-value	0.272	0.448	0.678	0.094
	Spearman's rho	-0.330	-0.075	0.026	-0.467
	p-value	0.077	0.799	0.920	0.092
TCI – HA TOT	*Pearsons's r*	0.035	0.152	0.427	-0.467
	p-value	0.904	0.573	0.060	0.119
	Spearman's rho	0.143	0.429	0.267	-0.678**
	p-value	0.658	0.126	0.285	0.008
TCI – RD1	*Pearsons's r*	-0.636*	0.058	-0.396	-0.491
	p-value	0.026	0.830	0.084	0.053
	Spearman's rho	-0.647*	0.218	-0.376	-0.272
	p-value	0.023	0.454	0.124	0.346
TCI – RD2	*Pearsons's r*	**-0.947*****	**-0.514***	0.146	0.468
	p-value	**< 0.001**	**0.031**	0.563	0.067
	Spearman's rho	**-0.971*****	**-0.505**	0.170	0.598*
	p-value	**< 0.001**	**0.065**	0.499	0.024
TCI – RD3	*Pearsons's r*	-0.560	-0.180	0.273	-0.006
	p-value	0.058	0.537	0.244	0.983
	Spearman's rho	-0.500	-0.055	0.301	0.018
	p-value	0.098	0.852	0.266	0.950
TCI – RD TOT	*Pearsons's r*	-0.907***	-0.220	0.036	0.020

	p-value	< 0.001	0.412	0.887	0.955
	Spearman's rho	-0.943***	-0.357	0.151	0.178
	p-value	<0 .001	0.210	0.551	0.452
**TCI – P**	*Pearsons's r*	-0.042	-0.769***	0.303	0.686**
	p-value	0.888	<0 .001	0.195	0.003
	Spearman's rho	-0.042	-0.661**	0.410	0.568*
	p-value	0.888	0.010	0.091	0.034
**TCI – SD1**	*Pearsons's r*	0.157	0.251	-0.288	0.355
	p-value	0.592	0.349	0.218	0.178
	Spearman's rho	0.131	0.134	-0.129	0.400
	p-value	0.685	0.649	0.606	0.156
**TCI – SD2**	*Pearsons's r*	0.036	0.342	0.475*	0.429
	p-value	0.903	0.194	0.034	0.097
	Spearman's rho	-0.029	0.436	0.650**	0.272
	p-value	0.928	0.119	0.004	0.346
**TCI – SD3**	*Pearsons's r*	-0.044	0.179	-0.054	0.506*
	p-value	0.881	0.508	0.820	0.045
	Spearman's rho	-0.135	0.223	0.218	0.489
	p-value	0.675	0.444	0.385	0.076
**TCI – SD4**	*Pearsons's r*	0.830***	0.585*	0.133	0.032
	p-value	< 0.001	0.028	0.575	0.906
	Spearman's rho	0.714**	0.679*	-0.117	-0.143
	p-value	0.009	0.008	0.643	0.627
**TCI – SD5**	*Pearsons's r*	0.721**	-0.019	0.195	0.322
	p-value	0.004	0.943	0.411	0.224
	Spearman's rho	0.725**	0.324	-0.077	0.108
	p-value	0.008	0.258	0.763	0.713
**TCI – SD TOT**	*Pearsons's r*	0.618*	0.391	0.157	0.336
	p-value	0.019	0.134	0.509	0.203
	Spearman's rho	0.667*	0.595*	0.243	0.108
	p-value	0.018	0.025	0.332	0.713
**TCI – SC1**	*Pearsons's r*	0.151	0.162	0.442	-0.085
	p-value	0.606	0.580	0.051	0.753
	Spearman's rho	0.116	-0.145	0.547*	0.091
	p-value	0.720	0.620	0.019	0.757
**TCI – SC2**	*Pearsons's r*	-0.209	0.215	0.435	0.232
	p-value	0.474	0.424	0.071	0.424
	Spearman's rho	0.029	0.306	0.219	0.234
	p-value	0.929	0.287	0.382	0.421
**TCI – SC3**	*Pearsons's r*	-0.695**	-0.049	0.229	-0.215
	p-value	0.006	0.857	0.331	0.424
	Spearman's rho	-0.463	-0.116	0.177	-0.116
	p-value	0.130	0.924	0.481	0.694
**TCI – SC4**	*Pearsons's r*	0.263	0.776***	0.235	-0.237
	p-value	0.408	<0 .001	0.318	0.378
	Spearman's rho	0.147	0.826***	0.034	-0.257
	p-value	0.648	<0 .001	0.893	0.376
**TCI – SC5**	*Pearsons's r*	-0.463	-0.258	0.576*	0.469
	p-value	0.096	0.373	0.012	0.067
	Spearman's rho	-0.339	-0.075	0.468	0.374
	p-value	0.280	0.799	0.050	0.088
**TCI – SC TOT**	*Pearsons's r*	-0.197	0.204	0.456*	0.062
	p-value	0.499	0.450	0.044	0.820
	Spearman's rho	-0.145	0.270	0.351	0.234
	p-value	0.653	0.350	0.153	0.421
**TCI – T1**	*Pearsons's r*	0.165	0.581*	-0.324	-0.922***

	p-value	0.572	0.018	0.164	< 0.001
	Spearman's rho	0.174	0.414*	-0.324	-0.954***
	p-value	0.589	0.141	0.164	< 0.001
**TCI – T2**	*Pearsons's r*	-0.200	0.016	-0.376	-0.568*
	p-value	0.494	0.952	0.102	0.022
	Spearman's rho	-0.265	-0.168	-0.518*	-0.355
	p-value	0.406	0.565	0.028	0.213
**TCI – T3**	*Pearsons's r*	-0.200	0.151	**-0.530***	**-0.790*****
	p-value	0.534	0.697	**0.016**	**< 0.001**
	Spearman's rho	-0.145	0.151	**-0.580***	**-0.666****
	p-value	0.653	0.697	**0.012**	**< 0.009**
**TCI – T TOT**	*Pearsons's r*	-0.013	0.320	**-0.460***	**-0.877*****
	p-value	0.969	0.264	**0.041**	**< 0.001**
	Spearman's rho	-0.029	0.107	**-0.433**	**-0.749****
	p-value	0.930	0.715	**0.072**	**< 0.002**

* p < .05, ** p < .01, *** p < .001.

S6. Ophthalmological evaluation of patient group

Twenty patients affected by GO (all female; mean age: 43 ± 16 years) and ten healthy subjects (all female; mean age: 38 ± 18 years) were enrolled in this study. Both groups were homogeneous for gender and age and their demographical characteristic (Table S1).
Control group (HC) showed normal values of peripheral thyroid and absence of serological TRAb. Normal visual acuity, values of bulbar proptosis (15.2 ± 1.3 mm) and normal ocular evaluation were also observable in HC. Specific ocular findings of the GO patient group are reported in Table 1.
The subgroup analysis (by CAS, group I: CAS<3, n=8; group II: CAS≥3, n=12) was performed.
A correlation between CAS and proptosis value was found (Pearson's r = 0.755; p<0.001), and consequently, GO patients from group II show significant higher proptosis (proptosis =20.9 ± 1.8 mm) respect to Group I patients (proptosis = 17.9 ± 1.9 mm, p=0.005).

Table S6. Demographical and ocular findings of the study population.

**Variable**	**GO (n=20)**
Age, y (mean±SD)	43±16
Gender	20 female
Smokers N.(%)	10/20 (50%)
GD duration (months)	14.9±4.6
Peripheral thyroid function:<br>• Euthyroid (without therapy) N. (%)<br>• Euthyroid (ongoing therapy) N. (%)<br>• Hyperthyroid (with therapy) N. (%)	 2 (10%)<br>16 (80%)<br>2 (10%)
TRAb titer (mean±SD) U/liter	5.4±2.6
Duration of GO (months)	10.4±1.6
**Ophthalmological evaluation**	
Visual acuity (mean±SD)	1±0
Proptosis mm	19.1±2.4 (range 16-23 mm).
CAS (mean±SD)	2.1±1.52 (range 0-5)
CAS≥3 N. (%)	8 (40%)

**Severity assessment**	
Mild N. (%)	18 (90%)
Moderate to severe N. (%)	2 (10%)
Presence of diplopia N (%)	2/20 (10%)

Table S7. Summary of CANTAB neuropsychological tasks employed (modified by [57])

CANTAB tasks	*Description*	*Performance indices*
Visual memory Paired Associates Learning (PAL) [54]	Episodic memory and learning. It requires subjects to learn the location of 1, 2, 3, 6, and 8 visual pattern-location pairings. For participants who fail to complete all stages, an adjusted total is calculated that allows for errors predicted in the stages that were not attempted	PAL Total errors (adjusted), PAL Total errors (six shapes, adjusted), PAL means of Tentative and PAL First trial memory score
Spatial Working Memory (SWM) [55]	Working memory capacity and strategy use. It requires subjects to "search through" boxes on the screen to find individual "blue tokens" without returning to a box where one had previously been found	SWM Between errors (Total) (returning to a box where a token has previously been found), the SWM Between errors (four boxes), (six boxes), and (eight boxes), and the SWM Strategy
Rapid Visual Information Processing (RVP) [56]	Sustained attention. It requires subjects to detect infrequent three- digit sequences from among serially presented digits	RVPA' (sensitivity to the target) and RVP Mean latency

Table S8. Temperaments and Character Inventory (TCI) – Dimensions of Personality Traits

Temperaments	*Subscales*	***Characters***	*Subscales*
Novelty Seeking (NS)	Exploratory excitability (NS1)<br>Impulsiveness (NS2)<br>Extravagance (NS3)[3]<br>Disorderliness (NS4)	*Self-Directedness (SD)*	Responsibility Vs. Blaming (SD1)<br>Purposefulness Vs. Lack Of Goal Direction (SD2)<br>Resourcefulness Vs. Inertia (SD3)<br>Self-Acceptance Vs. Self-Striving (SD4)<br>Congruent Second Nature Vs. Incongruent Habits (SD5)
Harm Avoidance (HA)	Anticipatory worry (HA1)<br>Fear of uncertainty (HA2)<br>Shyness/Shyness with strangers (HA3)<br>Fatigability/Fatigability and asthenia (weakness) (HA4)	*Cooperativeness (CO)*	Social acceptance vs. intolerance (C1)<br>Empathy vs. social disinterest (C2)<br>Helpfulness vs. unhelpfulness (C3)<br>Compassion vs. revengefulness (C4)<br>Principles vs. self-advantage (C5)
Reward Dependence (RD)	Sentimentality (RD1)<br>Openness to warm communication or social sensitivity (RD2)<br>Attachment (RD3)<br>Dependence on approval by others (RD4)	*Self-Transcendence (ST)*	Self-forgetful vs. self-conscious experience (ST1)<br>Transpersonal identification vs. self-isolation (ST2)<br>Spiritual acceptance vs. rational materialism (ST3)
Persistence (PS)			
